# The active form of the influenza cap-snatching endonuclease inhibitor baloxavir marboxil is a tight binding inhibitor

**DOI:** 10.1016/j.jbc.2021.100486

**Published:** 2021-02-27

**Authors:** Brendan Todd, Egor P. Tchesnokov, Matthias Götte

**Affiliations:** 1 Department of Medical Microbiology and Immunology, University of Alberta, Edmonton, Alberta, Canada; 2 Li Ka Shing Institute of Virology at University of Alberta, Edmonton, Alberta, Canada

**Keywords:** baloxavir marboxil, baloxavir acid, influenza A, influenza B, influenza polymerase, cap-snatching endonuclease, RNA-dependent RNA polymerase, viral replicase, BXA, baloxavir acid, BXM, baloxavir marboxil, FluB-ht, Influenza B Polymerase heterotrimer, nt, nucleotide, RdRp, RNA-dependent RNA polymerase, WT, wild-type

## Abstract

Baloxavir marboxil (BXM) is an FDA-approved antiviral prodrug for the treatment of influenza A and B infection and postexposure prophylaxis. The active form, baloxavir acid (BXA), targets the cap-snatching endonuclease (PA) of the influenza virus polymerase complex. The nuclease activity delivers the primer for transcription, and previous reports have shown that BXA blocks the nuclease activity with high potency. However, biochemical studies on the mechanism of action are lacking. Structural data have shown that BXA chelates the two divalent metal ions at the active site, like inhibitors of the human immunodeficiency virus type 1 (HIV-1) integrase or ribonuclease (RNase) H. Here we studied the mechanisms underlying the high potency of BXA and how the I38T mutation confers resistance to the drug. Enzyme kinetics with the recombinant heterotrimeric enzyme (FluB-ht) revealed characteristics of a tight binding inhibitor. The apparent inhibitor constant (*K*_i_^app^) is 12 nM, while the I38T mutation increased *K*_i_^app^ by ∼18-fold. Order-of-addition experiments show that a preformed complex of FluB-ht, Mg^2+^ ions and BXA is required to observe inhibition, which is consistent with active site binding. Conversely, a preformed complex of FluB-ht and RNA substrate prevents BXA from accessing the active site. Unlike integrase inhibitors that interact with the DNA substrate, BXA behaves like RNase H inhibitors that compete with the nucleic acid at the active site. The collective data support the conclusion that BXA is a tight binding inhibitor and the I38T mutation diminishes these properties.

Influenza virus infection is a source of significant morbidity and mortality worldwide (1, 2). Recent estimates suggest that Influenza infection is responsible for 300,000–650,000 deaths annually (2). Hence, there is a concern that the cocirculation of influenza, severe acute respiratory syndrome coronavirus 2 (SARS-CoV-2), and other respiratory viruses could exceed health-care capacity in several settings (3). Furthermore, the utility of anti-influenza therapies is often diminished due to the emergence of resistant strains (4). For example, amantadine, which antagonizes the influenza A proton channel, is no longer recommended as a treatment due to widespread resistance (4). Alternatively, neuraminidase inhibitors such as oseltamivir can be used for the treatment of influenza A and B (4); however, the potential clinical benefits are still debated (4). Recently, baloxavir marboxil (BXM), a first-in-class antiviral targeting the viral polymerase gained FDA approval (5). Intracellular hydrolysis of the prodrug yields baloxavir acid (BXA), which is the active form. BXM treatment, much like oseltamivir, results in a ∼20–25% reduction in time until symptom resolution and the clinical utility of both drugs is compromised by the emergence of resistant viruses (6, 7, 8). However, BXM can be given as a single dose while oseltamivir is given twice daily over a period of 5 days (6, 7). A recent study also demonstrated that a single-dose of BXM showed postexposure prophylactic efficacy in reducing household transmissions from 13.6% in the placebo group to 1.9% in the BXM group (9). Based on this data, the FDA expanded the approval to postexposure prevention.

Progress has also been made in elucidating the mechanism of inhibition of viral replication by BXA. The viral replication complex is a heterotrimer (ht), possessing a 7-methylguanylate (m^7^G) cap binding domain (PB2) that allows binding of cellular mRNAs (10, 11, 12), an endonuclease domain (PA) that cleaves the bound mRNA to generate primers for viral transcription in a process referred to as “cap-snatching” (11, 12, 13, 14, 15, 16), and the PB1 domain, which contains the RNA-dependent RNA polymerase (RdRp) active site that is required for RNA synthesis during replication and transcription (12, 15, 17, 18). PA recruits two divalent metal ions to its active site (14, 19), and BXA was designed to bind to these catalytic metal ions (19). A similar approach was utilized for the development of inhibitors of the human immunodeficiency virus type 1 (HIV-1) integrase (20, 21) and ribonuclease H (RNase H) inhibitors (22, 23). While several HIV-1 integrase inhibitors are approved for clinical use, HIV-1 RNase H inhibitors remain investigational. A common motif for these metal binders is three strategically positioned heteroatoms that form an “anchor domain” (24). Crystal structures of the truncated PA subunit with and without BXA have shown that the inhibitor binds to the two metal ions with three oxygen atoms and a hydrophobic area contributing to this binding (19). This area is generally referred to as “specificity domain,” which provides selectivity for a specific target (Fig. 1).

**Figure 1:**
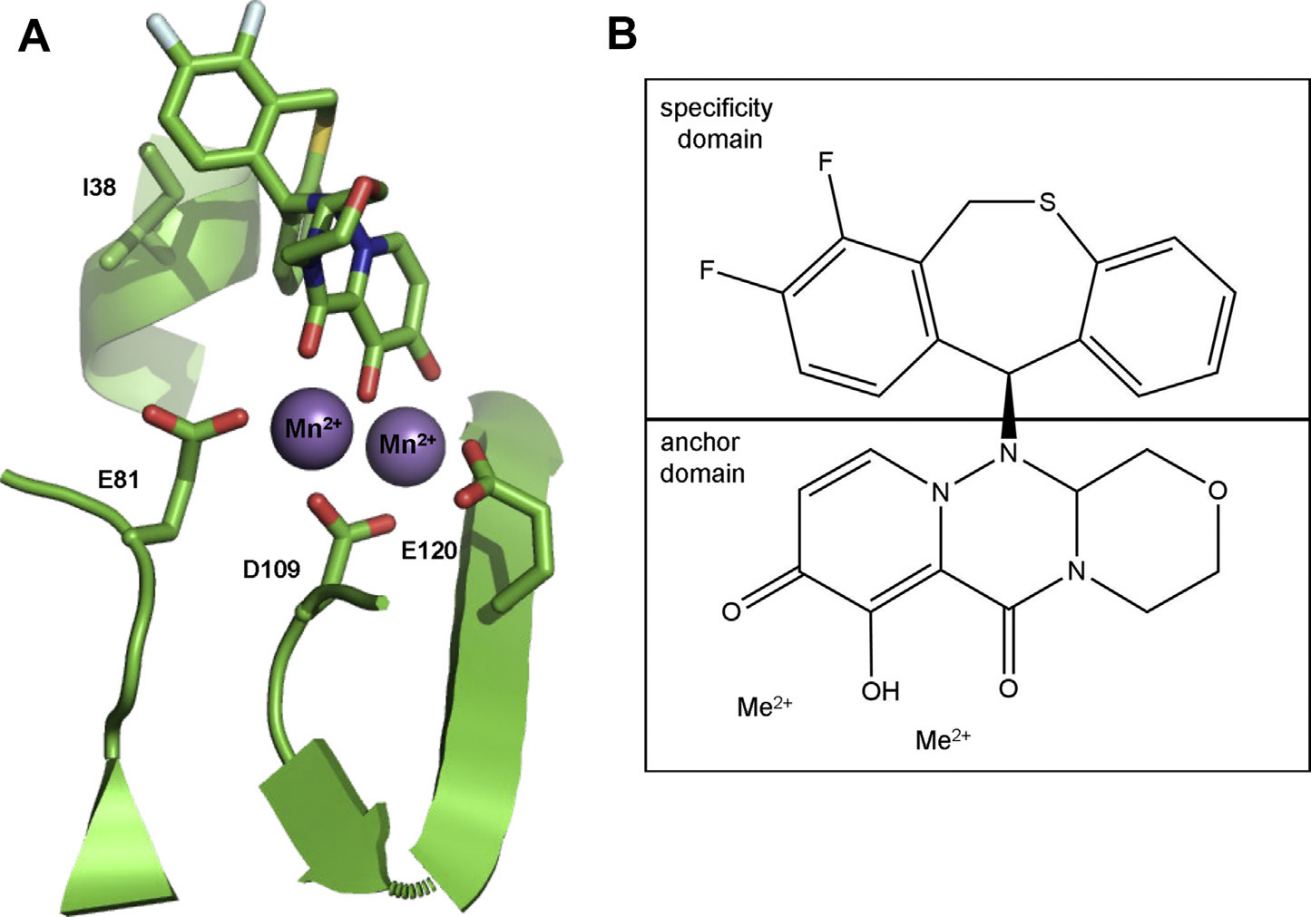
**Baloxavir acid (BXA) chelates divalent metal ions at the active site of the Influenza B cap-snatching endonuclease subunit (PA).***A*, model depicting baloxavir acid (BXA, *top*) binding to the Influenza B PA active site (PDB:6FS8) (21). Selected residues are depicted as sticks. E81, D109, and E120 coordinate metal ions (*purple balls*), which bind to the anchor domain of baloxavir. I38 forms part of the hydrophobic interface that interacts with the specificity domain of BXA. *B*, schematic diagram of baloxavir acid (BXA) highlighting anchor and specificity domains. Me^2+^ represents either Mg^2+^ or Mn^2+^.

Mutations conferring resistance to BXM have been observed clinically (6, 7, 8, 9, 19). In a recent trial, I38T/M/F substitutions appeared in 9.7% of patients receiving BXM (6). The impact of these substitutions on BXM susceptibility has been explored using cell culture systems. Mutant viruses are associated with up to 50-fold increases in EC_50_ values with I38T showing the most significant effect (19). The I38T mutation reduces van der Waals interactions between the hydrophobic area of BXA and its binding pocket (19, 25). Here we studied BXA-mediated inhibition of recombinant influenza B (FluB) heterotrimeric PA/PB1/PB2 RNA-dependent RNA polymerase (RdRp), referred to hereafter as FluB-ht. The biochemical data demonstrate that BXA can be classified as a tight binding inhibitor and that the I38T mutation diminishes these properties.

## Results

### Cap-snatching endonuclease activity of FluB-ht wild-type (WT) and FluB-ht PA I38T

**Figure 2:**
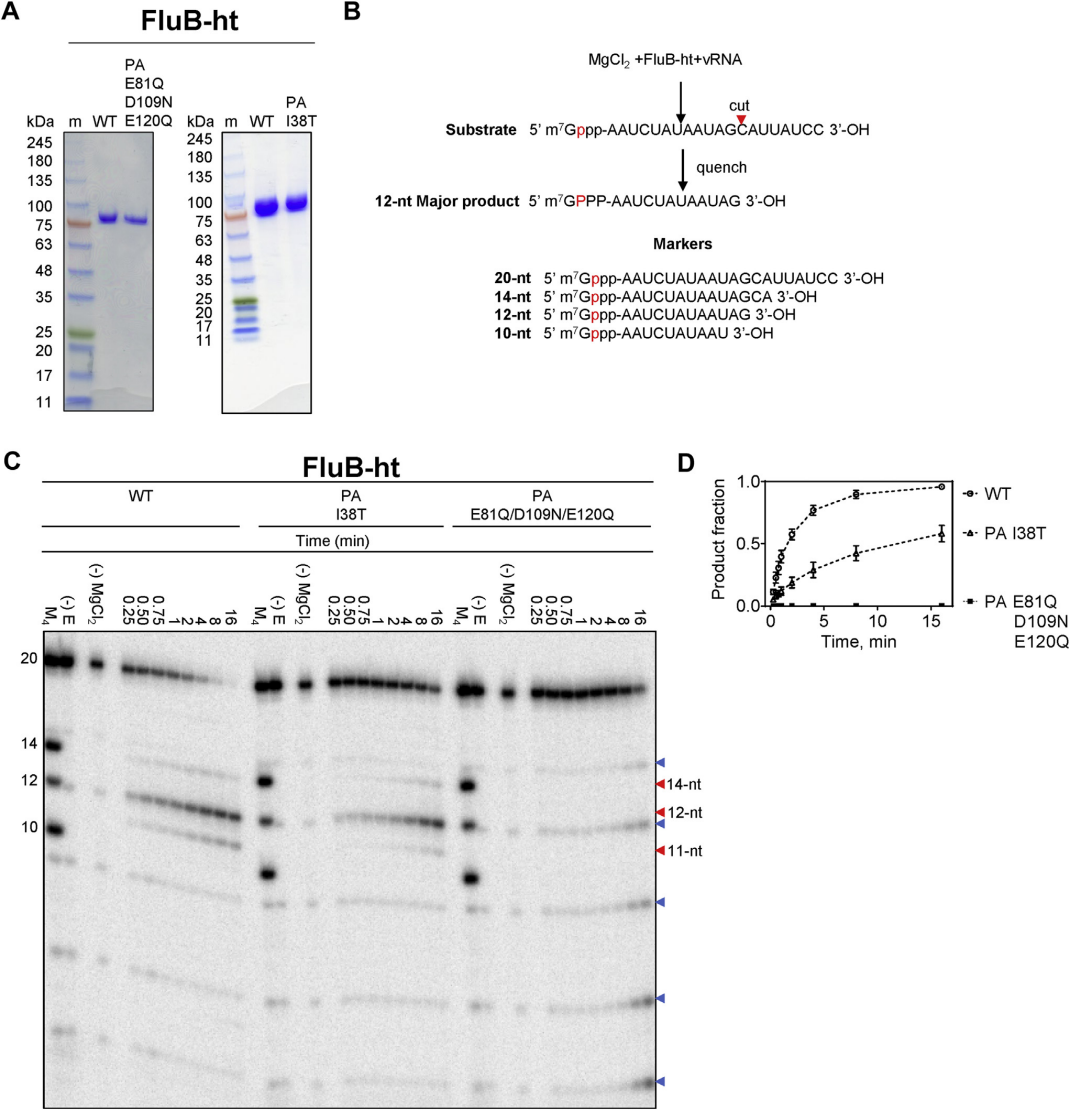
**Purification and biochemical characterization of the Influenza B polymerase heterotrimer (FluB-ht).***A*, SDS-PAGE migration patterns of the purified WT FluB-ht, baloxavir resistance mutant (PA I38T), or endonuclease-deficient mutant (PA E81Q/D109N/E120Q). Enzyme preparations were stained with Coomassie brilliant blue R-250. The size of the molecular weight markers (m) is indicated in kDa to the left of the gel. The band above the 75 kDa marker corresponds to full-length FluB-ht containing PA, PB1, and PB2 subunits. The identity of each of the subunits was confirmed *via* LC-MS/MS. *B*, schematic representation of the nuclease reaction with the position of the radiolabel highlighted in *red*. The sequences of the 20, 14, 12, and 10-nt markers are highlighted (Lane M_4_). *C*, endonuclease activity of WT, PA I38T, or endonuclease-deficient (PA E81Q/D109N/E120Q) FluB-ht on PAGE. M_4_ indicates the migration patterns of an equimolar mixture of 5’- m7G capped 20-nt, 14-nt, 12-nt, and 10-nt oligos here utilized as molecular weight markers (–) E indicates the migration pattern of 5’capped 20-nt substrate in the absence of enzyme. (–) MgCl_2_ indicates the results of the reaction after 16 min in the absence of Mg^2+^. *Blue arrows* indicate hydrolysis products while *red arrows* indicate the position of bona fide endonucleolytic cleavages. *D*, graphical representation of data shown in A. Data points are connected using a *dotted line* to illustrate the progress of the reaction. Product fraction refers to the ratio of the signal produced by the 5’ capped 11- and 12-nt products to the sum of these products plus the remaining substrate. The 14-nt product was not quantified as its contribution to overall signal was negligible. Error bars represent the standard deviation of at least three independent experiments. All experiments were performed under the following final conditions: 55 nM FluB-ht, 100 nM 20-nt substrate, 30 mM Tris-pH 7.5, 25 mM NaCl, 1.7 μM vRNA, and 5 mM MgCl_2_.

The baculovirus expression system has been successfully used to express the RdRp complex of several segmented negative sense RNA viruses including human influenza B (15, 17, 26, 27). Here we used this approach to generate WT FluB-ht, a variant associated with BXM resistance (FluB-ht PA I38T), and a variant containing a catalytically inactive endonuclease (FluB-ht PA QNQ) where metal coordinating residues were mutated (E81Q, D109 N, E120Q) (19, 28). The three complexes were purified to near homogeneity (Fig. 2*A*). Due to the similar molecular weight of the three domains comprising FluB-ht (PA, PB1, and PB2), the complex is seen as a single band on SDS PAGE (Fig. 2*A*). Diluting the sample helps to visualize three individual bands (26). In this study, the presence of all three peptides was confirmed using LC-MS/MS.

To evaluate the PA-mediated endonuclease activity of FluB-ht, we used an experimental design similar to that which has been previously described (15, 17, 29). The substrate for the endonuclease reaction is a radiolabeled capped 20-nt long RNA. It has been shown that efficient cleavage of the substrate requires the presence of a second RNA molecule, referred to as vRNA, which resembles the bound viral RNA (15, 29, 30, 31, 32, 33). Time-course experiments with each of the three enzymes are shown in Figure 2C. The cap-snatching reaction is initiated in the presence of Mg^2+^, which yields a 12-nt major product with cleavage after 5’G. Minor products represent a shorter 11-nt and a longer 14-nt RNA both with cleavage after 5’A. The three products are distinct from RNA background hydrolysis seen in the absence of enzyme. These control reactions show a faint hydrolysis product that migrates slightly faster than the major 12-nt product of the nuclease reaction. Differences in migration patterns are expected as nuclease products contain a 3’-hydroxyl group, while hydrolysis yields a negatively charged 3’-phosphate group. The FluB-ht PA I38T shows a very similar pattern as seen with the WT FluB-ht (Fig. 2*C*); however, a quantitative comparison of WT and PA I38T FluB-ht reveals a reduction of the overall activity of the PA I38T FluB-ht as has been noted by others (Fig. 2*D*) (19). Finally, the active site mutant, FluB-ht PA QNQ, shows a complete loss of endonuclease activity (Fig. 2, *C* and *D*). This data rules out the presence of contaminating endonucleases, while the hot spots for hydrolysis are seen with all three enzyme preparations and controls in the absence of enzyme.

### Inhibition of FluB-ht WT and FluB-ht PA I38T endonuclease by BXA

**Figure 3:**
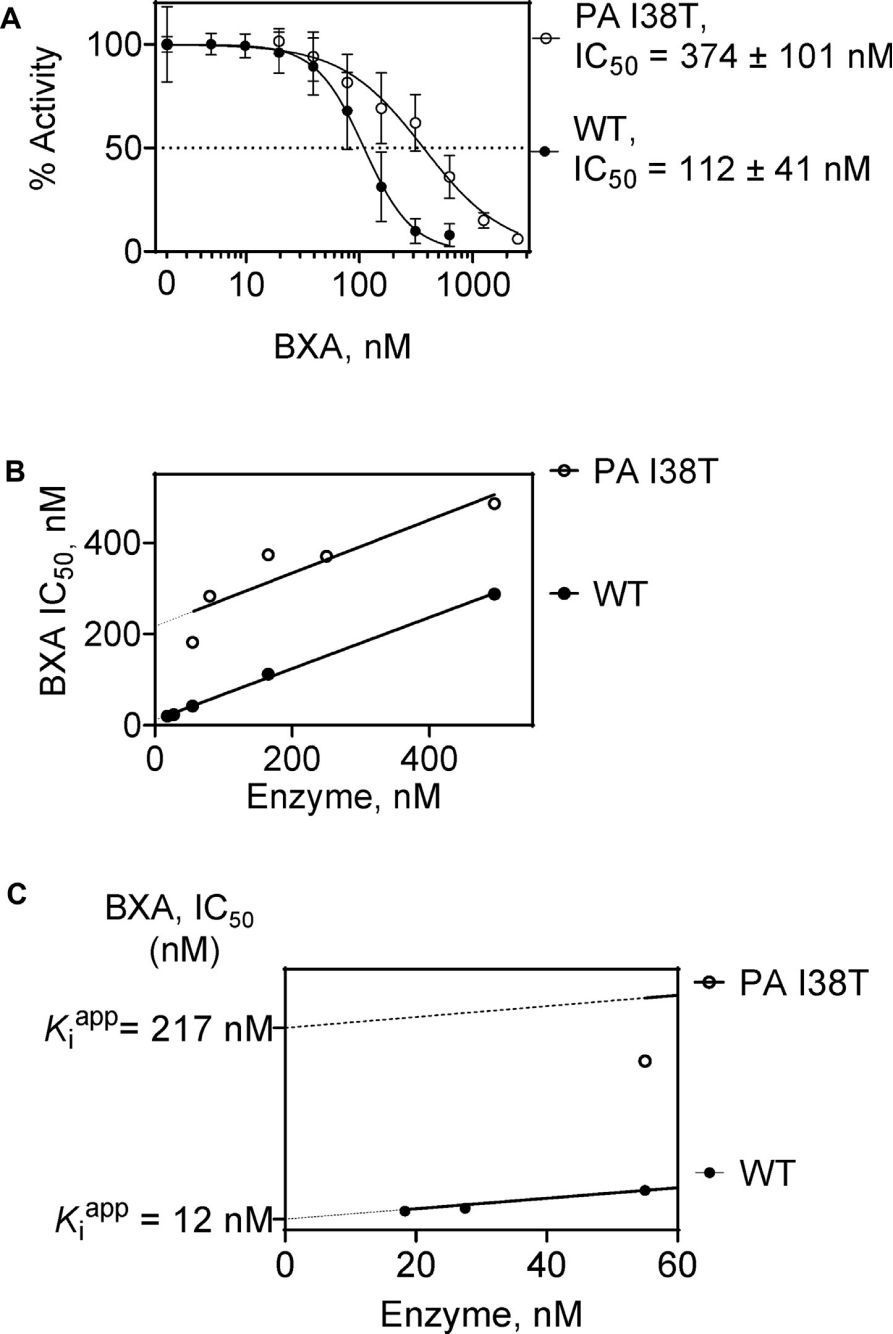
**Differential inhibition of WT and PA I38T FluB-ht by BXA.***A*, 165 nM of WT or PA I38T was mixed with increasing concentrations of BXA and inhibition quantified. The *dotted line* represents the half-maximal inhibitory concentration (IC_50_) of BXA. Error bars represent the standard deviation of at least three independent experiments. *B*, dependence of the IC_50_ value on FluB-ht concentration. Plots of IC_50_ versus enzyme concentration for both WT and PA I38T yield a linear relationship with a slope of approximately 0.5 (WT = 0.56, PA I38T = 0.58.) (*C*) Close-up of the Y-axis from *panel B* emphasizing the apparent inhibition constant (*K*i^app^) of BXA for WT and PA I38T FluB-ht. The *dotted line* indicates the threshold below which robust IC_50_ determination was not possible due to insufficient endonuclease activity. Percent error for WT IC_50_ measurements was 27% while percent error for PA I38T was 36%. All experiments were performed under the following final conditions: 100 nM 20-nt substrate, 30 mM Tris-pH 7.5, 5% DMSO, 25 mM NaCl, BXA as specified, FluB-ht as indicated, 1.7 μM vRNA, and 5 mM MgCl_2_.

To quantify inhibition of WT and PA I38T FluB-ht by BXA, we initially determined IC_50_ values of 112 nM and 374 nM for the WT and I38T variants, respectively (Fig. 3*A*). This represents an approximately threefold decrease in inhibition with the mutant enzyme. However, this IC_50_ value for WT FluB-ht is an order of magnitude higher than previously reported (5). The concentrations of enzyme used in this earlier study were not indicated, which makes it difficult to compare the data (5). The IC_50_ value of 112 nM measured in our experiments is similar to the enzyme concentration of 165 nM used in our assay. IC_50_ values for tight binding inhibitors are expected to increase with enzyme concentration (34). Specifically, plots of IC_50_ as a function of enzyme concentration correspond to the linear function IC_50_ = ½ [E] + *K*_i_^app^ (34).

To test this hypothesis, IC_50_ values were determined for both WT and PA I38T FluB-ht at various concentrations of enzyme (Fig. 3*B*). Plots of IC_50_ as a function of enzyme concentration for both WT and PA I38T FluB-ht yielded linear plots with slopes of ∼0.5 (Fig. 3*B*), suggesting tight binding in both cases. However, the *K*_i_^app^ of BXA for PA I38T of 217 nM is ∼18-fold increased with respect to WT FluB-ht (12 nM) (Fig. 3*C*). The lower this value, the higher the probability that tight binding is occurring. The ideal tight binding inhibitor would have an extrapolated *K*_i_^app^ of zero resulting in the IC_50_ being equal to exactly half the enzyme concentration. Furthermore, this 18-fold difference in *K*_i_^app^ is significantly larger than the initially observed threefold difference in IC_50_ that we obtained utilizing a single FluB-ht concentration (165 nM) illustrating the utility of this approach for the biochemical characterization of BXA and tight binding inhibitors in general. The observed IC_50_ values were independent within a narrow range of substrate concentrations (Fig. S1), which shows that the enzyme concentration is the only relevant variable that affects determination of IC_50_ values.

### Effects of order of addition on inhibition of FluB-ht WT and FluB-ht PA I38T by BXA

**Figure 4:**
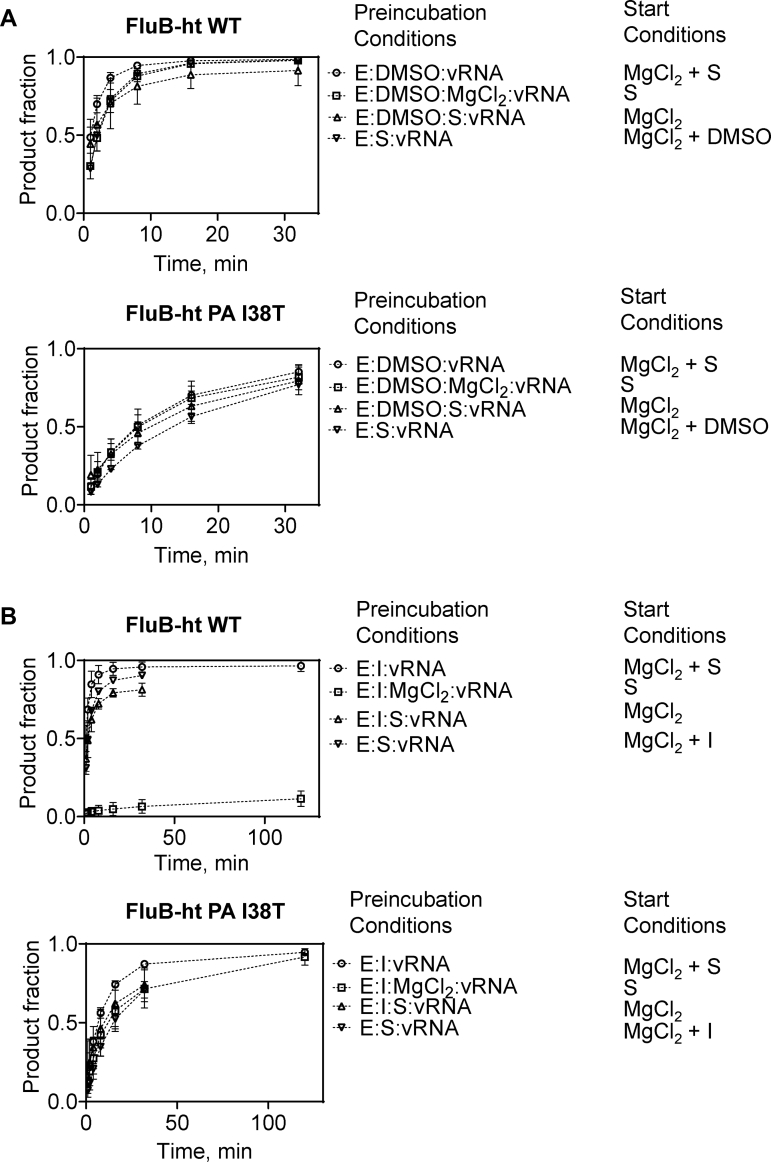
**Effects of the order of addition of FluB-ht (E), substrate (S), BXA (I), and MgCl**_**2**_**on the endonuclease activity of WT and PA I38T FluB-ht.***A*, order of addition of reaction components in the absence of BXA has negligible effects on reaction progress for both WT (*top*) and PA I38T (*bottom*) FluB-ht. *B*, Preincubation E, I, and MgCl_2_ is required for inhibition of WT (*top*) endonuclease activity. Inhibition of PA I38T (*bottom*) is not observed regardless of the order of addition the reaction components. Data represent at least three independent experiments. Error bars were omitted for clarity, Figure 4 with error bars is provided in the supplemental information (Fig. S2). All experiments were performed under the following final conditions: 100 nM 20-nt substrate, 30 mM Tris-pH 7.5, 5% DMSO, 25 mM NaCl, 75 nM BXA, 55 nM FluB-ht, 1.7 μM vRNA, and 5 mM MgCl_2_.

Previous structural studies have shown that BXA binds to the active site of the isolated PA endonuclease domain (19). However, these complexes lack the RNA substrate. Structures of FluB-ht with bound inhibitor and RNA are also not available. For cleavage to occur, the RNA must traverse the active site in close proximity to bound metal ions. Hence, RNA binding can prevent or reduce inhibitor binding. To test this hypothesis, we performed time-course assays where the order of addition of inhibitor and substrate was varied. The endonuclease reaction in the absence of inhibitor for both WT and PA I38T FluB-ht was largely unaffected by the order of addition of FluB-ht (E), capped 20-nt Substrate (S), and Mg^2+^ (Fig. 4*A*). In the presence of 75 nM BXA (I), inhibition was only observed under conditions where an E:I:Mg^2+^ complex was preformed (Fig. 4*B*). Conversely, under the same conditions no inhibition of the PA I38T FluB-ht variant was observed (Fig. 4*B*). Taken together, these data suggest that BXA binds to the PA endonuclease active site and prevents, at least locally, binding of RNA substrate. Nuclease activity was negligible over longer periods of time, suggesting slow dissociation of the inhibitor.

### Effect of BXA preincubation on inhibition of FluB-ht WT and FluB-ht PA I38T

**Figure 5:**
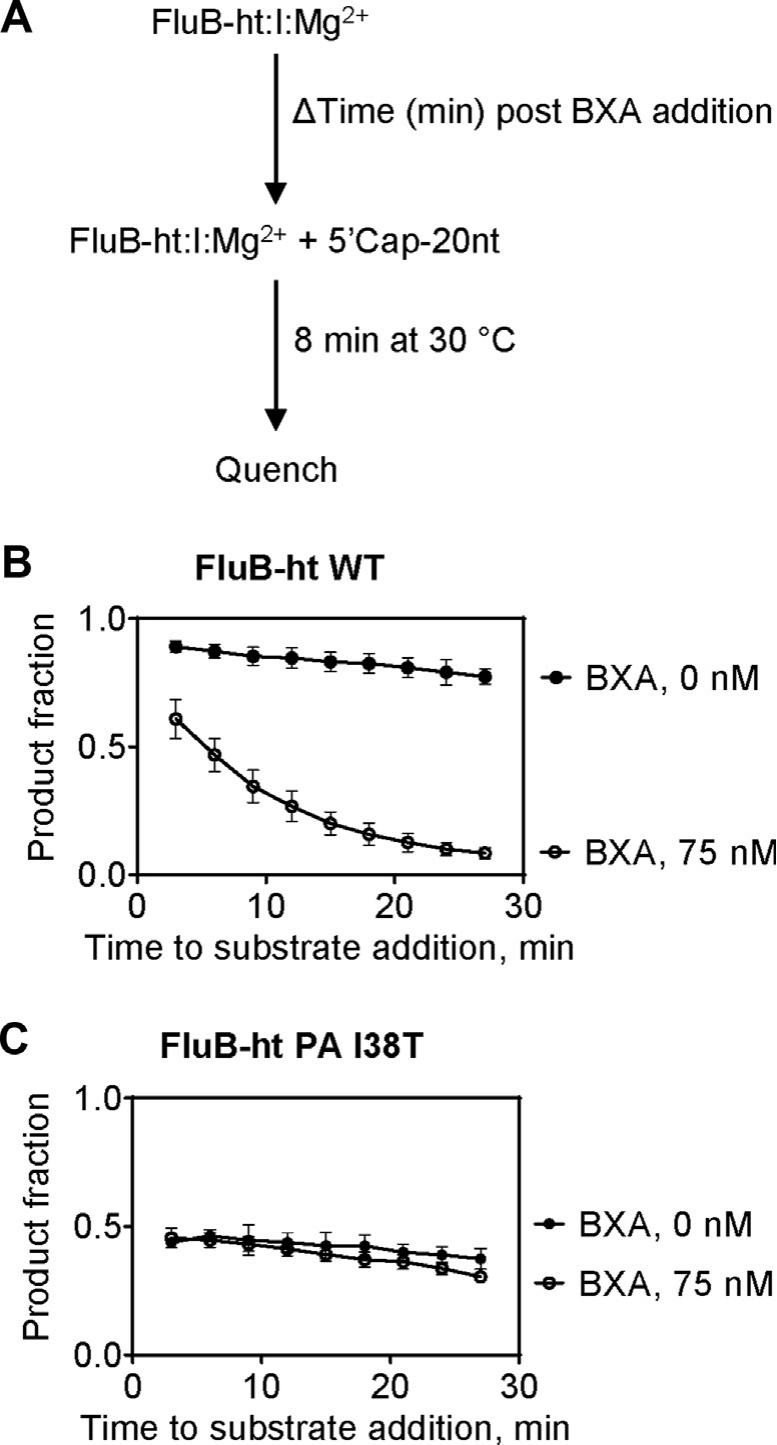
**Time-dependent inhibition of the PA endonuclease activity of FluB-ht by BXA (I).***A*, schematic representation of the experimental setup. *B*, inhibition of WT FluB-ht by BXA increases with the duration of BXA preincubation (*C*) Inhibition of PA I38T FluB-ht by BXA is time-independent. Error bars represent the standard deviation of at least three independent experiments. All experiments were performed under the following final conditions: 100 nM 20-nt substrate, 30 mM Tris-pH 7.5, 5% DMSO, 25 mM NaCl, BXA as specified, 55 nM FluB-ht, 1.7 μM vRNA, and 5 mM MgCl_2_.

To this end, WT or PA I38T FluB-ht, and BXA were preincubated for a fixed period of time with 75 nM BXA to allow formation of an E:I:Mg^2+^ complex before initiating the reaction with substrate. To assess the association kinetics of the inhibitor, we added the capped RNA substrate at different times of E:I:Mg^2+^ pre-incubation (Fig. 5*A*). For WT FluB-ht, increasing inhibition was observed as a function of preincubation length with maximal inhibition being observed after ∼30 min of preincubation (Fig. 5*B*). These data suggest that the association of BXA with PA is slow as is often the case for inhibitors that exhibit tight binding properties (34). Conversely, preincubation length had only negligible effects on the activity of the PA I38T FluB-ht (Fig. 5*C*). Furthermore, an effect is only observed when 600 nM BXA (eightfold more than for WT) is used (Fig. S3). The difference observed between WT and PA I38T FluB-ht provides additional evidence to show that the association of BXA with FluB-ht is impaired by the PA I38T substitution.

## Discussion

BXM is a first-in-class inhibitor of the influenza cap-snatching endonuclease subunit of the RdRp complex. Clinical trials revealed reductions in viral load, a faster time of recovery as compared with the placebo group, and utility as postexposure prophylaxis (6, 7, 8, 9, 19). BXM is now approved in several countries for the treatment of uncomplicated influenza A and B infection. The drug is highly potent with EC_50_ values in the low nanomolar range with subtle increases for influenza B as compared with influenza A (5, 19, 35). Mutations at position I38, predominantly I38T, were shown to confer resistance to BXM *in vitro* and *in vivo* (8, 19). In this context, EC_50_ values can increase up to 50-fold for influenza A and up to tenfold for influenza B (19). Structural studies with the isolated PA endonuclease domain bound to the active form of the drug, *i.e.*, BXA, provided important insight on inhibitor binding (19).

Here we employed a biochemical approach to study mechanisms associated with the high potency of the drug and its reduction in the presence of I38T. The endonuclease reaction was monitored with purified FluB-ht WT and FluB-ht PA I38T in the presence of Mg^2+^ and a 20mer, capped model RNA. In contrast to the reported structural studies, this approach may help to better understand the role of the RNA substrate in drug binding and inhibition. We identified a 12-nt major cleavage product, which is consistent with previous reports (15). The FluB-ht PA I38T mutant shows the same pattern, albeit with reduced intensity when compared with WT. The reduction in enzyme activity can translate in diminished replication capacity, which is not unusual for resistant mutant viruses (19). Indeed, mutant strains with amino acid substitutions at position 38 display fitness deficits in the absence of drug (5, 19).

Kinetic parameters for BXA-mediated inhibition of the nuclease activity have not been determined previously. Instead, Noshi and colleagues reported IC_50_ values of ∼2 nM (5). The low IC_50_ value points to tight binding. For tight binding inhibitors, IC_50_ values increase with increasing enzyme concentration. Changes in IC_50_ values as a function of enzyme concentration correspond to the equation: IC_50_ = ½ [E] + *K*_i_^app^_,_ where *K*_i_^app^ represents an approximation of the true inhibitor constant Ki (34). The determination of *K*_i_^app^ values allows one to compare the efficacy of inhibitors against WT and mutant enzymes. We observed an 18-fold difference in *K*_i_^app^ between WT and I38T FluB-ht, while plots of IC_50_ as a function of enzyme concentration yielded in both cases linear plots with slopes of ∼0.5.

Measurements of IC_50_ and *K*_i_^app^ values do not reveal the mechanism of inhibition, and Michaelis–Menten kinetics may also not distinguish between noncompetitive, competitive, and uncompetitive inhibition, because the steady-state assumption is not valid for tight binders (Fig. S4) (34). To address this problem, we designed order-of-addition experiments and show that a preformed complex of enzyme and RNA substrate prevents inhibition of nuclease activity. Inhibition requires a preformed complex of enzyme and inhibitor in the presence of divalent, catalytic metal ions. Based on this data we conclude that the bound inhibitor prevents simultaneous binding of the RNA substrate. Our data further indicate that inhibitor dissociation and association are both slow processes, which is often seen with tight binding inhibitors. The described circumstances are reminiscent of the mechanism of action of HIV-1 RNase H inhibitors (22, 36, 37). The HIV-1 RNase H active site of reverse transcriptase (RT) also contains two divalent metal ions (38, 39). Several RNase H inhibitors that bind the two metal ions have been described (39, 40, 41). Overlaying structures of HIV-1 RT in complex with a representative RNase H inhibitor and its nucleic acid substrate, respectively, revealed that the RNA would clash with the bound inhibitor (42). Structures of the N-terminal domain of the FluA PA subunit with a bound RNA oligonucleotide and BXA, respectively, also provide evidence for a significant overlap of the two binding sites (43). Thus, BXA or potent RNase H inhibitors prevent RNA binding, at least locally at the nuclease active sites. However, the nucleic acid binding groove is in both cases large enough to allow partial binding of the RNA even in the presence of inhibitor. Structures of FluB-ht with bound RNA substrate in the groove between PB2 and PA are necessary to address this problem directly. Overall, the proposed competitive mechanism is in stark contrast to HIV-1 integrase inhibitors that use the DNA substrate for binding (44).

In conclusion, our data provide strong evidence to show that BXA is a tight binding inhibitor that competes with the RNA substrate at the active site of the influenza cap-snatching endonuclease. There are also several study limitations. Tight binding of small molecule inhibitors can involve two steps that comprise the initial complex formation and a subsequent conformational change that stabilizes the complex. The current data do not distinguish between one-step and two-step binding modes. Moreover, inhibitor dissociation and association were here measured only indirectly, which precluded the determination of rate constants. It will be interesting to see how the I38T mutation affects these parameters. Despite these limitations, the measurements of *K*_i_^app^ values enable a quantitative assessment of both cap-snatching endonuclease inhibitors and mutant enzymes with resistance conferring amino acid substitutions. The proposed mechanism of action of BXA guides future studies aimed at the development of inhibitors with improved properties.

## Experimental procedures

### Enzymes and nucleic acids

Wild-type (WT) and variant FluB-ht were expressed and purified as described previously (B/Memphis/13/03) (15, 26). All FluB-ht variants were generated by DNA synthesis and purchased from Genscript (Piscastaway, NJ, USA). The NCBI accession numbers of the source protein sequences are as follows: PA AAU94844, PB1 AAU94857, and PB2 AAU94870. The 5’-triphosphorylated RNAs (5’-pppRNA) used to generate the 5’ m^7^G capped oligos were designed according to Reich *et al.*, 2020 (15). 5’-pppRNAs of up to 20 nucleotides (nts) were used to generate capped oligos and purchased from ChemGenes (Wilmington, MA, USA) while a 39-nt RNA without triphosphorylation at the 5’ end corresponding to the influenza B promoter consensus sequence (vRNA) was purchased from Dharmacon (Lafayette, CO, USA) (15). The sequence of the RNA used to generate the longest (20 nt) capped oligo is 5’ ppp-AAUCUAUAAUAGCAUUAUCC 3’. The 14-nt, 12-nt, and 10-nt oligos are shorter versions of this 20-nt oligo that have been truncated at the 3’ end. Their sequences are as follows: 14-nt oligo, 5’ ppp-AAUCUAUAAUAGCA 3’, 12-nt oligo, 5’ ppp-AAUCUAUAAUAG 3’, 10-nt oligo 5’ ppp-AAUCUAUAAU 3’. The capped 20-nt oligo was used as a substrate for the endonuclease reactions, while an equimolar mix of capped 20-nt, 14-nt, 12-nt, and 10-nt oligos were used as molecular weight markers. The sequence of the vRNA is as follows:

5’AGUAGUAACAAGAGGGUAUUGUAUACCUCUGCUUCUGCU 3’. Capping and radiolabeling of RNA oligos were performed using the New England Biolabs Vaccinia Capping System (Fisher Scientific, Edmonton, Alberta, Canada). Briefly, [α-^32^P] GTP (PerkinElmer, Boston, MA, USA), RNA markers, and vaccinia capping enzyme were mixed and the reactions allowed to proceed for 30 min at 37 °C. Reactions were then incubated for 10 min at 95 °C to inactivate the capping enzyme. Capped, radiolabeled RNA oligos were then further purified using GE healthcare microspin G-25 columns (Chicago, IL, USA) followed by phenol chloroform extraction.

### Baloxavir acid (BXA)

BXA was purchased from MedChemExpress (Monmouth Junction, NJ, USA) and resuspended at a concentration of 10 mM in 100% DMSO.

### FluB-ht PA endonuclease assay

The influenza nuclease assay was performed as described previously by Reich *et al*., 2014 and the concentrations of WT and variant FluB-ht were modified (15). Briefly, 55 nM of WT or variant FluB-ht was incubated at 30 °C with 1.7 μM vRNA in 30 mM Tris-HCl pH 7.5, 25 mM NaCl, and 5 mM MgCl_2_ (buffer A). 25 mM NaCl was chosen based on a NaCl optimization experiment (Fig. S5). The reactions were then initiated with 100 nM of 20-nt substrate. At the indicated time points, aliquots of the reaction master mix were quenched with formamide and 25 mM EDTA. Reactions were heat-inactivated at 95 C° for 10 min and resolved on a 20% denaturing polyacrylamide gel. The reaction products were then visualized by phosphorimaging. Product fractions were determined using QuantityOne software (Biorad). Product fraction refers to the ratio of the signal produced by the 5’-capped 11- and 12-nt products to the sum of these products plus the remaining substrate. The 14-nt product was not quantified as its contribution to overall signal was negligible. The resulting data were plotted using GraphPad Prism 8 (Graphpad, San Diego, California) (36).

### IC_50_ determination for inhibition of the FluB-ht PA endonuclease by BXA

For IC_50_ determinations, various concentrations of FluB-ht WT or FluB-ht PA I38T were incubated with 1.7 μM vRNA and increasing concentrations of BXA at 30 °C in buffer A and 5% DMSO for 5 minutes. Reactions were then initiated with the 20-nt capped RNA substrate. Reactions were quenched at 12 min post initiation and the products resolved and visualized as described above. The resulting data was quantified as described for the endonuclease assay, normalized based on activity in the absence of inhibitor, graphed using Prism 8 (Graphpad, San Diego, CA, USA), and IC50 values were determined. Percent error was determined as follows: % error = (Standard Deviation/Mean IC50) ∗100%

### Time-dependent inhibition assay

In total, 55 nM of FluB-ht WT or FluB-ht PA I38T was incubated with 75 nM BXA, 5% DMSO and 1.7 μM vRNA in buffer A at 30 °C for 3, 6, 9, 12, 15, 18, 21, 24, or 27 min. Following this, the reactions were initiated *via* the addition of 20-nt capped, radiolabeled, RNA substrate and allowed to proceed for 8 min. At this time, the reactions were quenched, resolved, and visualized as described above.

### Order of addition assays

In total, 55 nM FluB-ht WT or FluB-ht PA I38T and 100 nM capped 20-nt substrate were mixed as specified with and without 75 nM BXA in buffer A with 5% DMSO and incubated at 30 °C for 1 hour to allow for complex formation. The reactions were then initiated and allowed to proceed for 1, 2, 4, 8, 16, 32, or 120 min. The products were then separated *via* 20% polyacrylamide denaturing gel electrophoresis (PAGE) in 1x TBE buffer and visualized by phosphorimaging.

## Data availability

All data are included within this article.

## Supporting information

This article contains supporting information (34).

## Conflict of interest

The authors have no conflicts of interest to declare.
