# Supplementary material for: The active form of the influenza cap-snatching endonuclease inhibitor baloxavir marboxil is a tight binding inhibitor
Source: J Biol Chem. 2021 Feb 27;296:100486. doi: 10.1016/j.jbc.2021.100486 (PMC8065212; doi:10.1016/j.jbc.2021.100486)
Supplement: Figures S1–S5 [file mmc1.docx]

Supporting Information for

**The active form of the influenza cap-snatching endonuclease inhibitor baloxavir marboxil is a tight binding inhibitor**

**Brendan Todd^1^, Egor P. Tchesnokov^1^ and Matthias Götte^1,2*^**

^1^Department of Medical Microbiology and Immunology, University of Alberta, Edmonton, Alberta, Canada. ^2^Li Ka Shing Institute of Virology at University of Alberta, Edmonton, Alberta, Canada.

^*^Corresponding author

E-mail: [gotte@ualberta.ca](mailto:gotte@ualberta.ca)

**Running title: Mechanism of action of Baloxavir**

**Keywords:** baloxavir marboxil, baloxavir acid, influenza A, influenza B, influenza polymerase, cap-snatching endonuclease, RNA-dependent RNA polymerase, viral replicase

Included materials:

**Supplementary Figure S1.** IC_50_ of baloxavir acid (BXA) as a function of substrate concentration.

**Supplementary Figure S2.** Figure 5 from the manuscript with error bars.

**Supplementary Figure S3.** Time dependent inhibition of FluB-ht I38T variant under increasing concentrations of baloxavir acid (BXA).

**Supplementary Figure S4.** Michaelis-Menten kinetics of baloxavir acid (BXA) inhibition.

**Supplementary Figure S5**. Effects of NaCl and KCl titration on FluB-ht endonuclease activity.


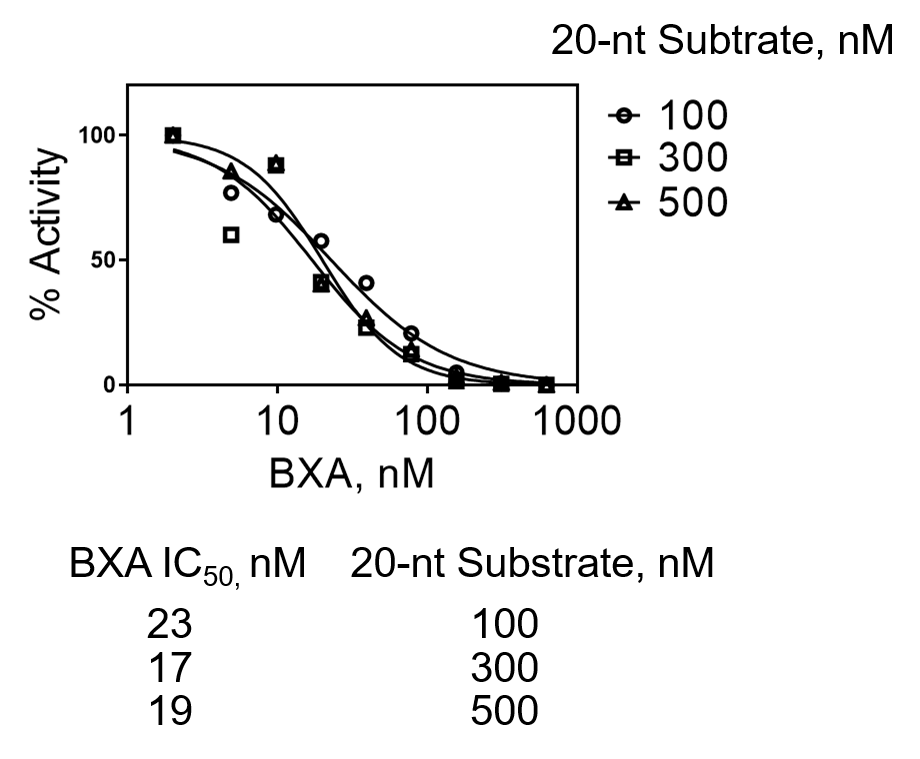


**Supplementary Figure S1.** IC_50_ of baloxavir acid (BXA) as a function of substrate concentration. The IC_50_ of baloxavir acid was measured in the presence of 100, 300, and 500 nM 20-nt radiolabeled capped substrate, 55 nM FluB-ht WT, 30 mM Tris-HCL pH 7.5, 25 mM NaCl, 5 mM MgCl_2_, and 1.7 µM vRNA.


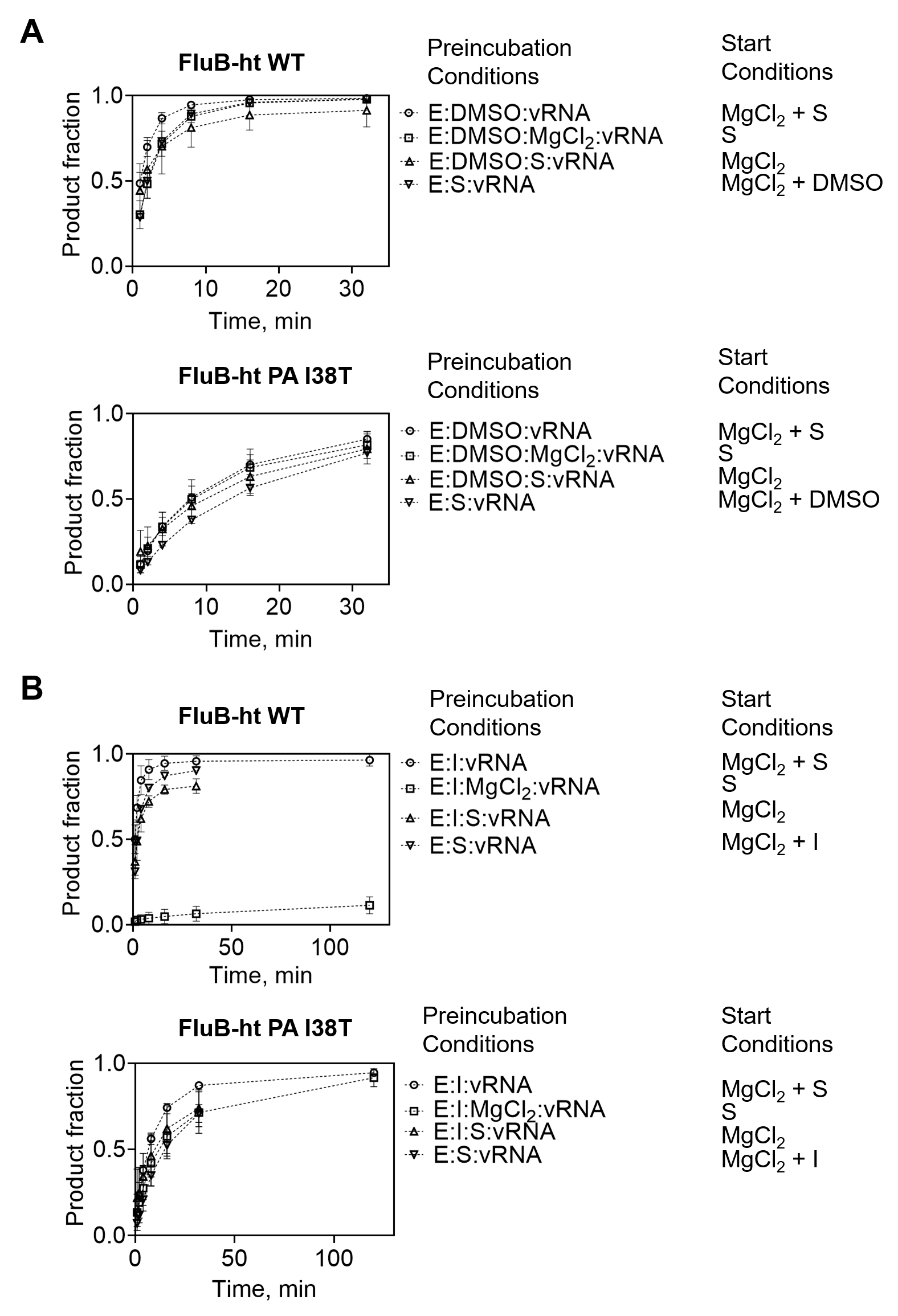


**Supplementary Figure S2.** Figure 5 from the manuscript with error bars. Error bars represent the standard deviation of at least three independent measurements. Final conditions: 30 mM Tris-HCL pH 7.5, 25 mM NaCl, 5 mM MgCl_2_, 55 nM FluB-ht WT or PA I38T, 1.7 µM vRNA, 5% DMSO, 75 nM BXA (I), and 100 nM 20-nt radiolabeled capped substrate.


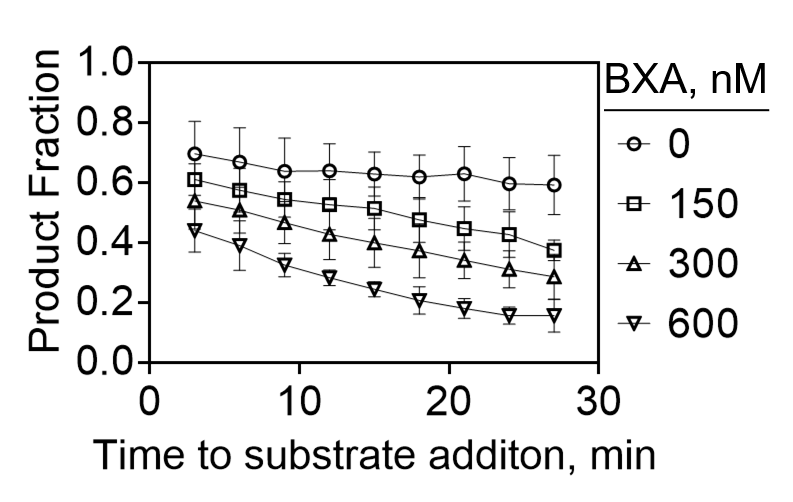


**Supplementary Figure S3.** Time dependent inhibition of FluB-ht I38T variant under increasing concentrations of baloxavir acid (BXA). Error bars represent the standard deviation of three independent experiments. Final conditions: 30 mM Tris-HCL pH 7.5, 25 mM NaCl, 5 mM MgCl_2_, 55 nM FluB-ht PA I38T, 1.7 µM vRNA, 5% DMSO, BXA as indicated and 100 nM 20-nt radiolabeled capped substrate.


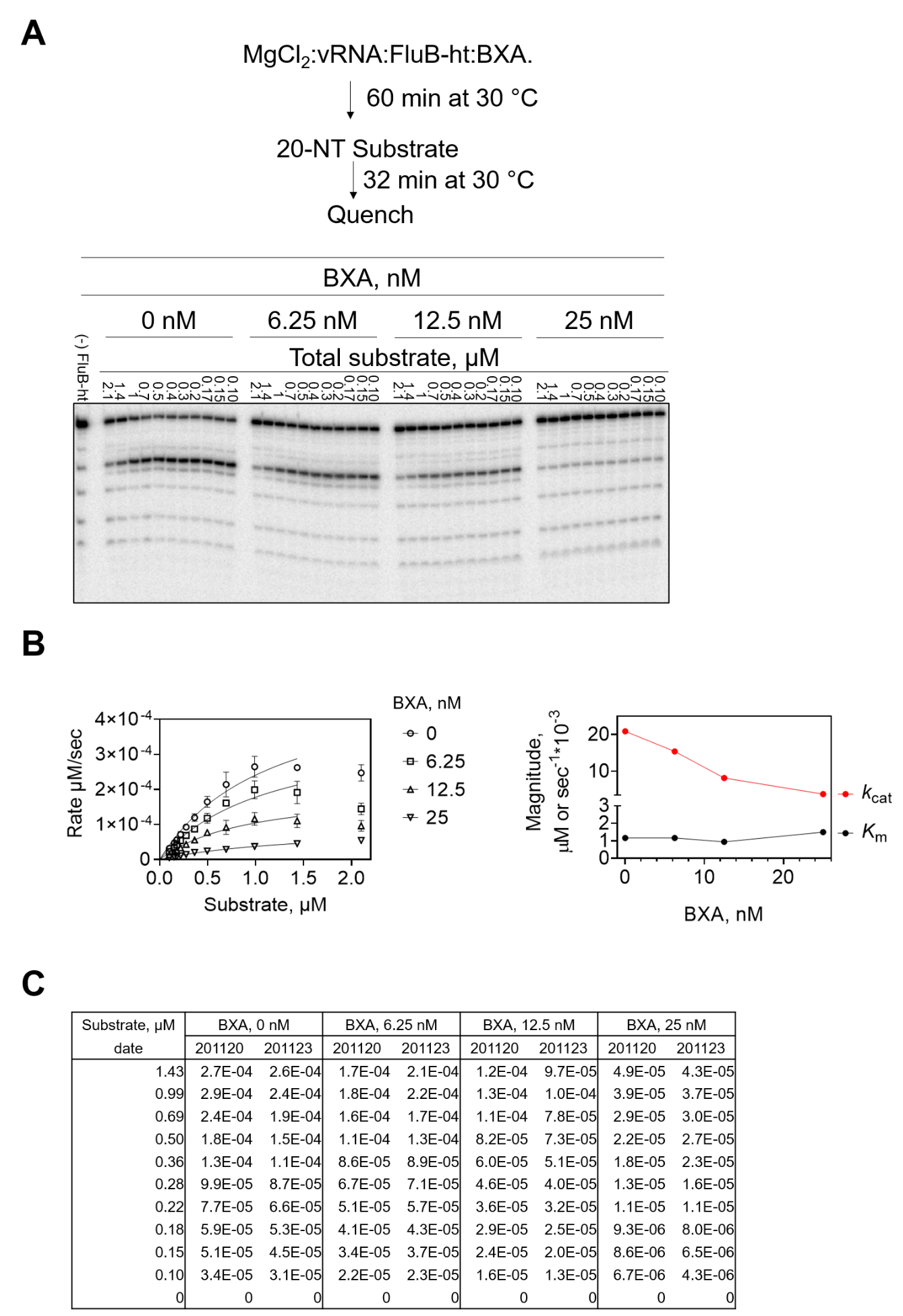


**Supplementary Figure S4.** Michaelis-Menten kinetics of BXA inhibition. (A) Scheme detailing the experimental setup and representative data showing the effect of BXA on substrate utilization by FluB-ht. (B) Graphic representation and analysis of the data presented in panel A are shown on the left-hand side graph. The effects of increasing concentrations of BXA on *k*_cat_ and *K*_m_ are illustrated on the right-hand side graph. Data represents the average of two independent experiments. The final reaction conditions were as follows: 30 mM Tris-HCL pH 7.5, 25 mM NaCl, 5 mM MgCl_2_, 25 nM FluB-ht, 1.7 µM vRNA, 5% DMSO, BXA as indicated and 20-nt capped substrate as indicated. The indicated substrate concentrations were achieved by mixing 0.1 µM 20-nt radiolabeled capped substrate RNA with increasing amounts of unlabeled substrate. Hence, at concentrations > 1.5 µM the labeled substrate is significantly outcompeted, which results in artificial rate reductions. The data point at 2.1 µM was therefore not considered for *k*_cat_ and *K*_m_ measurements. Although the reduction in *k*_cat_ and constant *K*_m_ values point to noncompetitive inhibition, this interpretation is not necessarily correct for tight binding inhibitors like BXA (34). Tight binding of enzyme and inhibitor depletes the concentration of free enzyme and the steady-state assumption is no longer valid. For these reasons, we designed order of addition experiments demonstrating that BXA indeed competes with the RNA substrate (Figure 4).


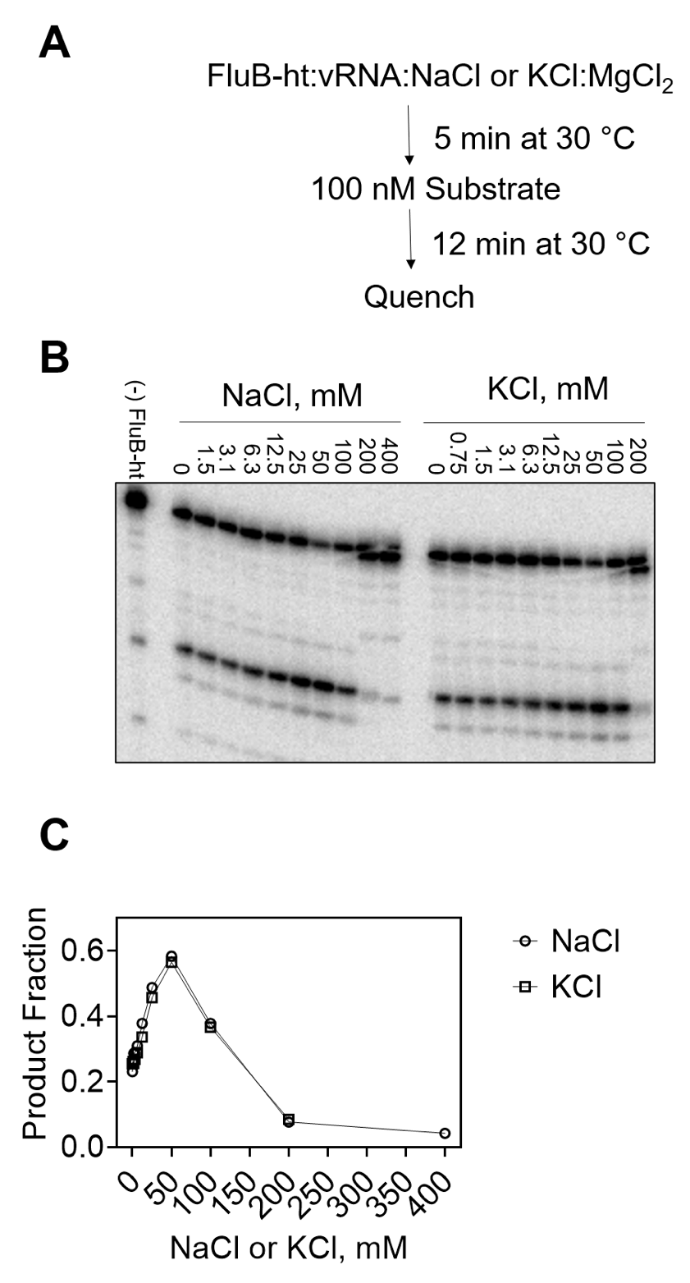


**Supplementary Figure S5**. Effects of NaCl and KCl titration on FluB-ht endonuclease activity. 55 nM FluB-ht, 1.7 µM vRNA, 5 mM MgCl_2,_ and either NaCl or KCl at the indicated concentrations were incubated for 5 minutes at 30 °C prior to the initiation of the reactions with 100 nM of substrate. (A) Schematic detailing the reaction setup. (B) PAGE migration pattern of the reaction products. (C) graphical representation of the data in B.
